# Fluorine-18 Labeled Fluorofuranylnorprogesterone ([^18^F]FFNP) and Dihydrotestosterone ([^18^F]FDHT) Prepared by “Fluorination on Sep-Pak” Method

**DOI:** 10.3390/molecules24132389

**Published:** 2019-06-28

**Authors:** Falguni Basuli, Xiang Zhang, Burchelle Blackman, Margaret E. White, Elaine M. Jagoda, Peter L. Choyke, Rolf E. Swenson

**Affiliations:** 1 Imaging Probe Development Center, National Heart, Lung, and Blood Institute, National Institutes of Health, Rockville, MD 20850, USA; 2 Molecular Imaging Program, National Cancer Institute, National Institutes of Health, Bethesda, MD 20892, USA; 3 Laboratory of Genitourinary Cancer Pathogenesis, National Cancer Institute, National Institutes of Health, Bethesda, MD 20892, USA

**Keywords:** fluorine-18, fluorination on Sep-Pak, [^18^F]FFNP, [^18^F]FDHT, mass spectrometry, molar activity

## Abstract

To further explore the scope of our recently developed “fluorination on Sep-Pak” method, we prepared two well-known positron emission tomography (PET) tracers 21-[^18^F]fluoro-16α,17α-[(R)-(1′-α-furylmethylidene)dioxy]-19-norpregn-4-ene-3,20-dione furanyl norprogesterone ([^18^F]FFNP) and 16β-[^18^F]fluoro-5α-dihydrotestosterone ([^18^F]FDHT). Following the “fluorination on Sep-Pak” method, over 70% elution efficiency was observed with 3 mg of triflate precursor of [^18^F]FFNP. The overall yield of [^18^F]FFNP was 64–72% (decay corrected) in 40 min synthesis time with a molar activity of 37–81 GBq/µmol (1000–2200 Ci/mmol). Slightly lower elution efficiency (~55%) was observed with the triflate precursor of [^18^F]FDHT. Fluorine-18 labeling, reduction, and deprotection to prepare [^18^F]FDHT were performed on Sep-Pak cartridges (PS-HCO_3_ and Sep-Pak plus C-18). The overall yield of [^18^F]FDHT was 25–32% (decay corrected) in 70 min. The molar activity determined by using mass spectrometry was 63–148 GBq/µmol (1700–4000 Ci/mmol). Applying this quantitative measure of molar activity to in vitro assays [^18^F]FDHT exhibited high-affinity binding to androgen receptors (K_d_~2.5 nM) providing biological validation of this method.

## 1. Introduction

**Figure 1:**
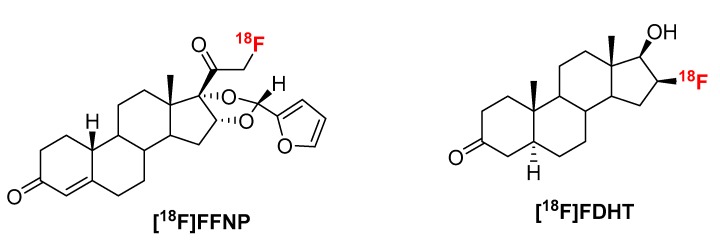
Structures of [^18^F]FFNP and [^18^F]FDHT.

Recently we have developed a novel method, “fluorination on Sep-Pak”, to prepare prosthetic groups for radiofluorination of biomolecules which requires neither azeotropic drying of fluorine-18 nor addition of base [1,2,3]. Hence, it is less time consuming and more suitable for base and temperature sensitive starting materials. Fluorine-18 was trapped on an anion exchange cartridge (PS-HCO_3_) and dried by flowing anhydrous acetonitrile. The direct incorporation of fluorine-18 was achieved by passing the precursor solution (0.5 mL/min) through this cartridge. Recently this method has been expanded to prepare a variety of fluorine-18 labeled compounds via a well-established copper-mediated radiofluorination [4]. Therefore we envisioned that “fluorination on Sep-Pak” method might be applicable to other room temperature fluorine-18 labeling reactions. To test the hypothesis, this radiolabeling method have been considered to prepare two well-established PET tracers (Figure 1), 21-[^18^F]fluoro-16α,17α-[(R)-(1′-α-furylmethylidene)dioxy]-19-norpregn-4-ene-3,20-dione furanyl norprogesterone ([^18^F]FFNP) and 16β-[^18^F]fluoro-5α-dihydrotestosterone ([^18^F]FDHT) which have been shown to have clinical relevance.

Breast cancer (BC) is the most common type of cancer in women worldwide [5,6,7]. Estrogen receptor alpha (ERα) and progesterone receptor (PR) are important steroid hormone receptor biomarkers used to determine prognosis and to predict benefit from endocrine therapies for breast cancer patients [6,7]. Advancement of early detection and treatment of breast cancer is the key parameter for the steady decrease of breast cancer mortality. Molecular imaging is a useful technique for early detection and quantitative measurement of disease both in primary and across metastatic sites of disease [8]. The radioligand [^18^F]FFNP demonstrates high relative binding affinity to PR and low nonspecific binding. It has been used for quantitative assessment of PR expression in vivo using PET [9,10,11]. Human safety and dosimetry have been assessed for identifying PR-positive breast cancer [12]. The literature reported procedure of radiolabeling to prepare [^18^F]FFNP is by a conventional radiolabeling method using azeotropically dried [^18^F]KF/K_222_/K_2_CO_3_ and a triflate precursor (1) in acetonitrile either at room temperature or at elevated temperature [13,14]. With this radiosynthetic requirement for a triflate precursor and the potential clinical applications, [^18^F]FFNP represented an ideal tracer for testing the “fluorination on Sep-Pak” method.

Prostate cancer (PC) is the most common cancer in men all over the world. It is the second leading cause of death from cancer [5]. Androgen receptor (AR) is a member of the nuclear receptor superfamily and it has a central role in prostate cancer progression [15,16]. Therefore, AR is the focus of detection and therapeutic treatment of PC [11,17,18]. PET imaging of AR can be an important tool to quantify expression in both primary tumors and metastatic sites simultaneously. It can also be a useful tool for evaluation of AR-targeted therapies. Fluorine-18 labeled 16β-fluoro-5α-dihydrotestosterone ([^18^F]FDHT, Figure 1) has been clinically proven useful for detection and relative quantification of AR expression [19,20,21,22]. Synthesis of [^18^F]FDHT was originally reported by reacting its triflate precursor with n-[^18^F]Bu_4_NF/n-Bu_4_NOH in THF at 55 °C [23]. In this procedure, LiAlH_4_ was used as reducing agent. Later the mild reducing agent, NaBH_4_, was used for better compatibility in an automated synthesis module [13]. Further improvements have been reported using a fully automated radiolabeling procedure [24]. Herein we report the radiosynthesis of [^18^F]FFNP and [^18^F]FDHT via the “fluorination on Sep-Pak” strategy. The purity was assessed by high-performance liquid chromatography (HPLC). The quality of the [^18^F]FDHT was further tested in vitro using saturation binding assays from which the affinity (K_d_) for AR was determined. 

## 2. Results and Discussion 

### 2.1. [^18^F]FFNP

**Scheme 1:**
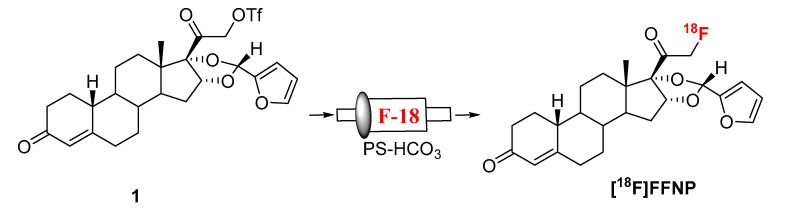
Synthesis of [^18^F]FFNP.

Synthesis of [^18^F]FFNP was first reported from its triflate precursor (**1**) with n-Bu_4_N[^18^F]F/n-Bu_4_NOH in THF at room temperature with a decay-corrected radiochemical yield (RCY) of 2–13% [9]. D. Zhou et al. observed a better RCY with the triflate precursor which was purified without aqueous workup [13]. Fluorination was achieved by using K[^18^F]F/K_222_/K_2_CO_3_ as a fluorinating agent for the same triflate precursor (**1**) in acetonitrile. Up to 77% RCY (isolated after HPLC, decay-corrected) of [^18^F]FFNP had been reported starting with 1.85 GBq (50 mCi) of [^18^F]fluoride in 90 min [13]. In these literature reported methods, fluorination was achieved at room temperature, therefore we envisioned that our recently developed “fluorination on Sep-Pak” method might be suitable to prepare [^18^F]FFNP. To test this hypothesis, an acetonitrile (0.5 mL) solution of the triflate precursor (1.5 mg) was passed through a PS-HCO_3_ cartridge (0.5 mL/min) containing [^18^F]fluoride followed by washing the cartridge with 1 mL acetonitrile. Over 40% of trapped fluoride was eluted from the cartridge (Scheme 1, Table 1).

**Figure 2:**
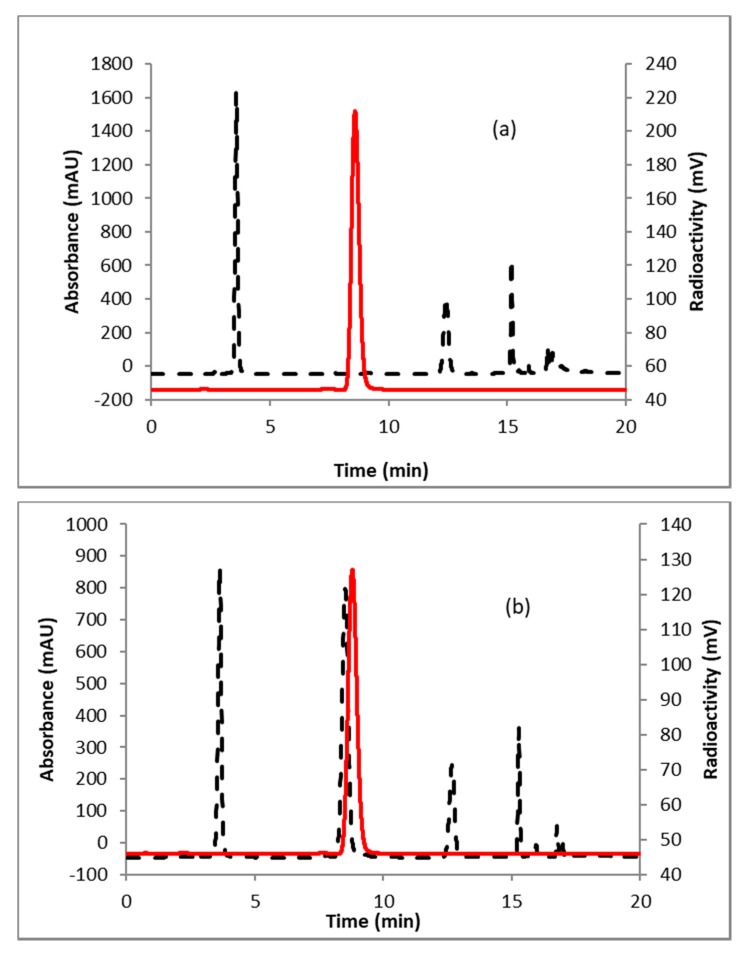
HPLC analysis (method A) of crude reaction mixture of (**a**) [^18^F]FFNP; (**b**) [^18^F]FFNP, co-injected with the non-radioactive standard. Solid line, in-line radio detector; dotted line, UV detector at 254 nm.

**Figure 3:**
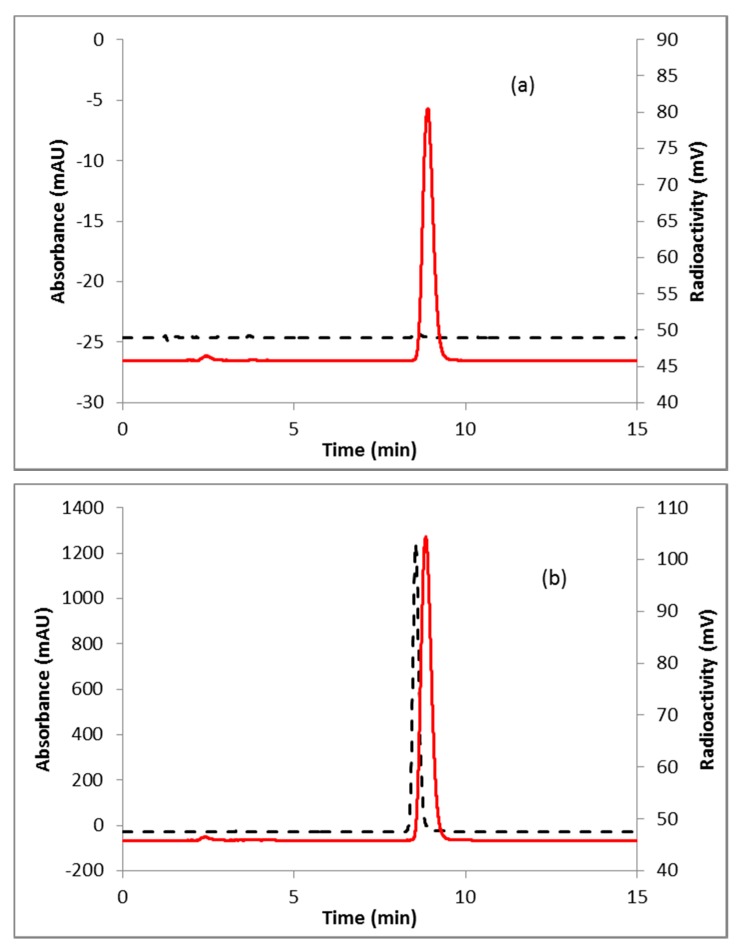
HPLC analysis (method A) of pure (**a**) [^18^F]FFNP; (**b**) [^18^F]FFNP, co-injected with the non-radioactive standard. Solid line, in-line radiodetector; dotted line, UV detector at 254 nm.

A representative HPLC of the crude product is shown in Figure 2a. The identity of the product was confirmed by co-injecting the crude reaction mixture with authentic nonradioactive standard, FFNP, (Figure 2b). Inspired by this result, the fluoride elution efficiencies were tested with a higher amount of precursor (Table 1) and over 70% elution efficiency was observed with 3 mg of the precursor. No significant improvement of the elution efficiency was noticed by further increasing the amount of precursor (5 mg). Literature methods used 2–4 mg of the precursor [13,14]. Fluoride incorporation efficiencies were also tested using different solvents (Table 1). Better elution of the product was observed with acetonitrile. After standardizing the elution efficiencies with a low amount of activity (0.37–0.74 GBq, 10–20 mCi) the full-scale reactions were performed with high amount (6.7–8.5 GBq, 180–230 mCi) of activities. The mixture was purified by HPLC using semi-preparative column (method B) to produce > 98% radiochemically pure (Figure 3a) product with a molar activity of 37–81 GBq/µmol (1000–2200 Ci/mmol, *n* = 6). The overall RCY was 64–72% (decay corrected, *n* = 6) in 40 min. The identity of the product was confirmed by comparing its HPLC retention time with co-injected authentic non-radioactive standard (Figure 3b). Using the same amount of precursor (3 mg) as reported in the literature, comparable RCY (64–72% vs. 77%) was obtained in a shorter synthesis time (40 min vs. 70 min). For a direct comparison of the RCY, synthesis of [^18^F]FFNP was performed using [^18^F]KF/K_2_CO_3_/K_222_ with 3 mg of triflate precursor (1) following the literature method [13]. In a typical reaction starting with 7.2 GBq (195 mCi) of [^18^F]fluoride, 2.0 GBq (55 mCi) of the product was obtained in 70 min (43% RCY, decay corrected). The lower RCY could be due to the aqueous workup of the triflate salt precursor as reported in the literature [13]. The workup procedure for the triflate salt in this experiment is unknown as this is from a commercial source.

### 2.2. [^18^F]FDHT

**Scheme 2:**
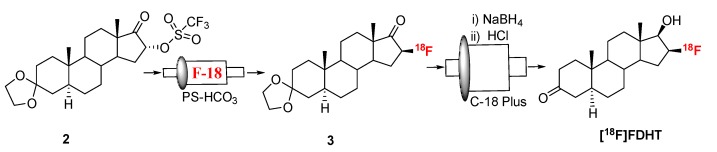
Synthesis of [^18^F]FDHT.

**Figure 4:**
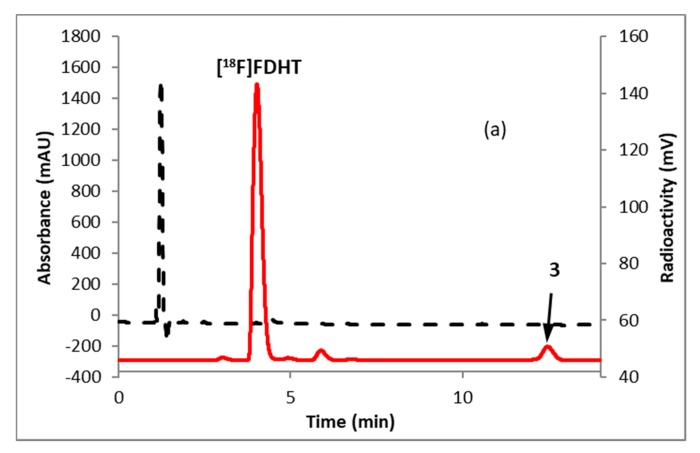

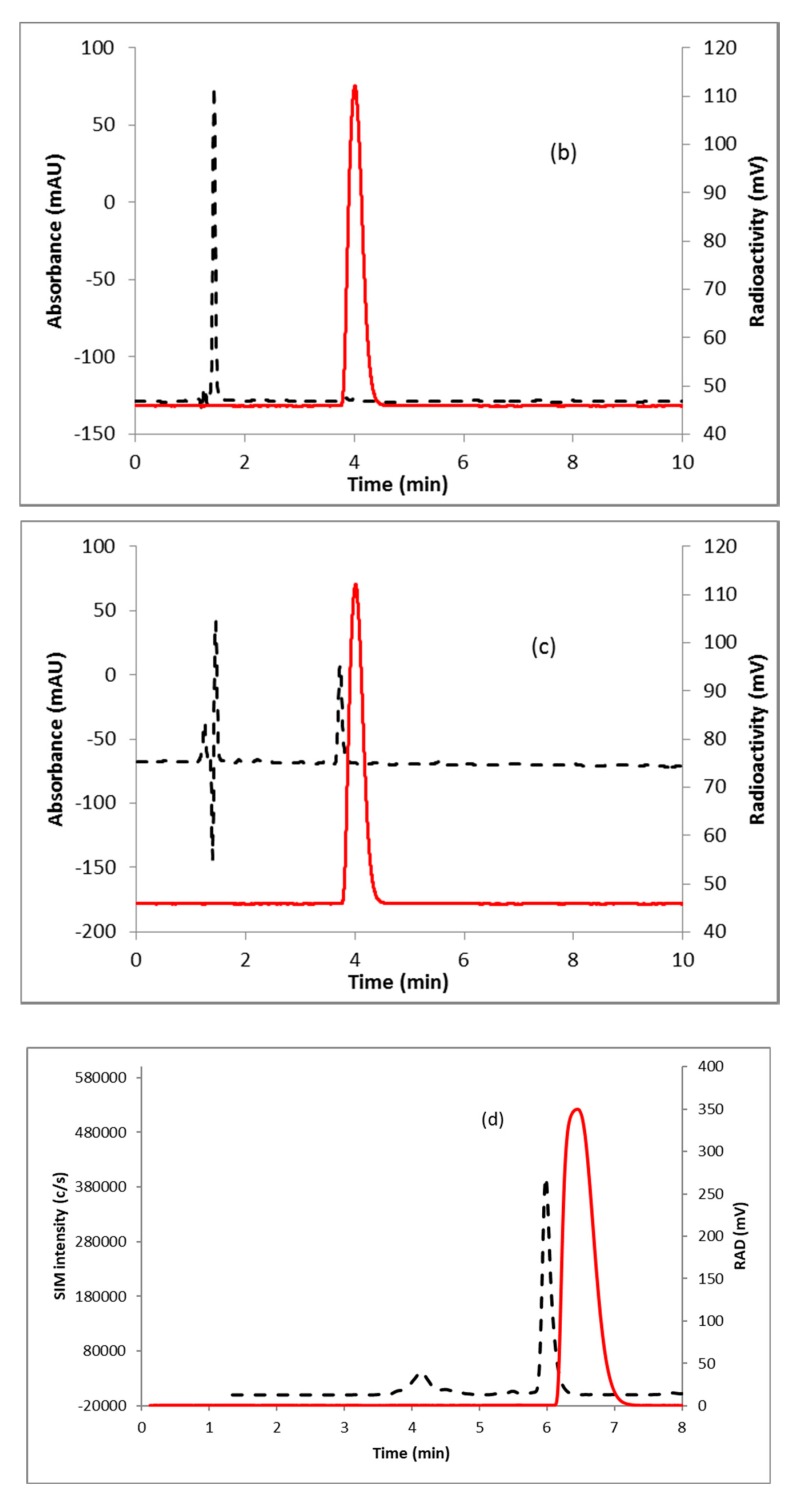
HPLC analysis (method A) of (**a**) crude reaction mixture of [^18^F]FDHT; (**b**) pure [^18^F]FDHT; (**c**) [^18^F]FDHT, co-injected with the non-radioactive standard; (**d**) LC/MS analysis (method E) of [^18^F]FDHT in SIM mode. Solid line, in-line radiodetector; dotted line, UV detector at 254 nm (for **a**–**c**); for d: dotted line, SIM intensity of *m*/*z* 350.

Fluorination efficiency to prepare intermediate **3** (Scheme 2) was similarly tested using different conditions (Table 2). The highest fluoride incorporation efficiency was obtained using 5 mg of precursor in 0.5 mL anhydrous tetrahydrofuran (THF) followed by washing the Sep-Pak with 1 mL acetonitrile. The next step was to reduce the intermediate **3**. The originally reported literature method used a strong reducing agent, LiAlH_4_ which had been successfully replaced by a milder reducing agent, NaBH_4,_ for better compatibility in the automated module [13,24]. However, it has been mentioned in the literature that the quenching of a large excess of NaBH_4_ with acetone is necessary, otherwise, it can further reduce the deprotected ketone produced during the acid deprotection step, resulting in significant decomposition of [^18^F]FDHT [13]. In this current method, the reduction step was performed on the Sep-Pak by passing (1 mL/min) through an aqueous solution of NaBH_4_ followed by water wash, which quantitatively reduced intermediate **3** and no quenching of NaBH_4_ with acetone was needed. The deprotection step was also performed on the Sep-Pak using 6N HCl. HPLC analysis (Figure 4a) of the crude reaction mixture indicated the clean conversion of intermediate **3** to the final product, [^18^F]FDHT. The final product was purified by HPLC on a semi-preparative column. The overall RCY was 25–32% (decay corrected, *n* = 12) in a 70 min synthesis time with a radiochemical purity of >98% by analytical HPLC (Figure 4b). The identity of the product was confirmed by comparing its HPLC retention time with co-injected, authentic non-radioactive standard, FDHT (Figure 4c). The identity of the product was further confirmed by comparing its mass associated with radiation peak (Figure 4d). The RCY (25–33%, *n* = 12) using the Sep-Pak method was comparable with the literature method in shorter synthesis times but used slightly higher amounts of precursor (5 mg vs. 4 mg). For a direct comparison of the RCY, the literature method was tested with 5 mg of precursor (**2**) [13]. In a typical reaction starting with 5.9 GBq (160 mCi) of [^18^F]fluoride, 1.8 GBq (49 mCi) (31%, decay corrected) of the product was obtained in 90 min.

To measure the molar activity (the measured radioactivity per mole of the compound), a calibration curve is typically generated by using UV absorption area of known concentration of non-radioactive standard. Using this calibration curve the mass of the labeled tracer is determined. Due to the very poor UV absorption of FDHT (Figure 4c), it was not possible to measure the molar activity by this method. Mass spectrometer connected to liquid chromatography (LC-MS) is a useful technique for quality control analysis of precursors and nonradioactive standards. The mass associated with the radioactive tracer is too low to be detected by a mass spectrometer commonly used for molecular mass determination. Recently our group demonstrated the use of liquid chromatography/tandem mass spectrometry (LC-MS/MS) for the optimization of fluorine-18 labeling reaction with fluorine-19 [25]. Coiller et al. developed an in-line concentration method by using a Sep-Pak to detect the mass of the tracers [26]. We used a similar approach to characterize and determine the molar activity of [^18^F]FDHT by using LC-MS system. The collected fraction of [^18^F]FDHT from the HPLC was trapped on the Sep-Pak and eluting with a minimum volume of ethanol (0.2 mL) and water (0.8 mL). A calibration curve was generated using the selected ion monitoring (SIM) of the Advion expression CMS system of a known quantity of nonradioactive standard FDHT. Mass of [^18^F]FDHT (known amount of radioactivity) was determined using this calibration curve. The molar activity determined by this method was 63–148 GBq/µmol (1700–4000 Ci/mmol, *n* = 12).

### 2.3. In Vitro Binding Assays

**Figure 5:**
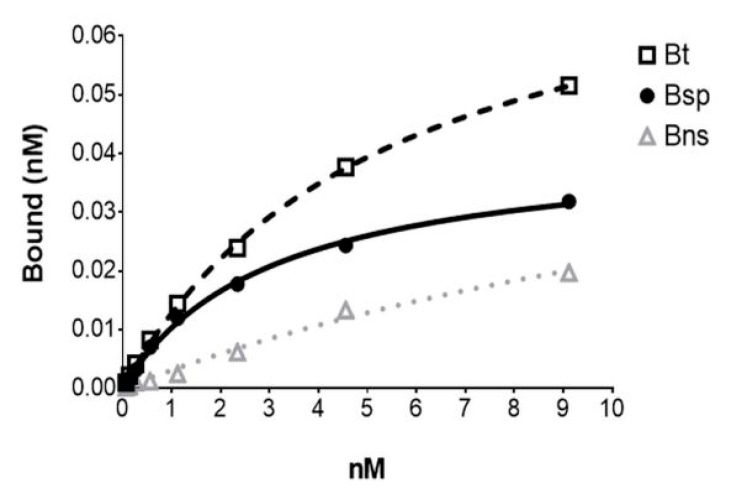
The representative plot from an in vitro [^18^F]FDHT saturation binding assay using. a DU145 AR+ tumor cytosol with each point representing the average of duplicates. B_t_ = B_total_; B_sp_ = B_specific_; B_ns_ = B_non-specific_ (determined in the presence of 10^−6^ M unlabeled FDHT).

[^18^F]FDHT exhibited high-affinity binding with a K_d_ of 2.49 ± 0.52 nM (mean ± SE; *n* = 6) determined from saturation binding studies using either DU145 AR+ (human androgen receptor) transfected cells or tumor cytosols prepared from DU145 AR+ xenograft mouse models (Figure 5). This K_d_ value compares favorably with the K_d_ of [^3^H]FDHT (2.5 nM) and IC_50_ of FDHT indicating the accuracy of the molar activity measurement with this method [22,27]. From the cell assays, the AR concentration (B_max_) was 2.97 ± 0.66 × 10^5^ receptors per cell (mean ± SE; *n* = 4) which was consistent with findings by Pandit-Taskar et al. [22]. The AR concentration in the tumor cytosols (B_max_) was found to be 0.170 ± 0.048 femtomoles/µg protein (mean ± SE; *n* = 2).

## 3. Materials and Methods

### 3.1. Materials, Chemicals, and Methods

Triflate precursor for the synthesis of [^18^F]FFNP was purchased from ChemShuttle (Hayward, CA, USA). Other precursors and non-radioactive standards were obtained from ABX GmbH (Radeberg, Germany). PBS 1X buffer (Gibco) was obtained from Life Technologies (Carlsbad, CA, USA). Normal saline was obtained from Quality Biological (Gaithersburg, MD, USA). PD10 MiniTrap^TM^ columns were obtained from GE Healthcare Bioscience (Pittsburg, PA, USA). All other chemicals and solvents were received from Sigma-Aldrich (St. Louis, MO, USA) and used without further purification. Fluorine-18 was obtained from the National Institutes of Health cyclotron facility (Bethesda, MD, USA). Chromafix 30-PS-HCO_3_ anion-exchange cartridge was purchased from Macherey-Nagel (Düren, Germany) and the packing material was reduced to half (~20 mg) for better elution efficiency of [^18^F]fluoride [3]. Phenomenex Luna C18 (2) column (10 × 250 mm, 5 µm) was purchased from Phenomenex (Torrance, CA, USA). All other columns and Sep-Pak cartridges used in this synthesis were obtained from Agilent Technologies (Santa Clara, CA, USA) and Waters (Milford, MA, USA), respectively. Sep-Pak C-18 Plus was conditioned with 5 mL ethanol, 10 mL air, and 10 mL water. Semi-prep HPLC purification and analytical HPLC analyses for radiochemical work were performed on an Agilent 1200 Series instrument equipped with multi-wavelength detectors. Mass spectrometry (MS) was performed on Advion expression compact mass spectrometer (CMS) with an ESI source (Ithaca, NY, USA). The LC inlet was Agilent 1200 series chromatographic system equipped with 1260 quaternary pump, 1260 Infinity autosampler, 1290 thermostatted column compartment, and the radiation detector. Column flow was split (1:4) between the mass spectrometer and the radiation detector. Instrument control and data processing were performed using Advion Mass Express and Quant Express Software.

HPLC conditions: Method A; Column: Agilent XDB C18 column (4.6 × 150 mm, 5 µm). Mobile phase: A: water (0.1% TFA); B: acetonitrile (0.1% TFA). Isocratic: 55% B; flow rate: 1 mL/min. Method B; Column: Phenomenex Luna C18 (2) column (10 × 250 mm, 5 µm). Mobile phase: A: water; B: acetonitrile. Isocratic: 55% B; flow rate: 4 mL/min. Method C; Column: Phenomenex Luna C18 (2) column (10 × 250 mm, 5 µm). Mobile phase: A: water; B: acetonitrile. Isocratic: 40% B; flow rate: 4 mL/min. Method D; Column: Agilent XDB C18 column (4.6 × 150 mm, 5 µm). Mobile phase: A: water (0.1% TFA); B: acetonitrile (0.1% TFA). Isocratic: 55% B; flow rate: 1 mL/min. Method E; Column: Agilent Poroshell 120 EC-C18 column (4.6 × 50 mm, 1.8 µm). Mobile phase: water (0.1% formic acid); B: acetonitrile (0.1% formic acid). Gradient: 5% B for 1 min, 5-95% B within 5.95 min and held at 95% B for 2 min, returned to initial conditions; flow rate: 1 mL/min. The column oven was kept at 40 °C throughout the analysis. The injection volume was 20 µL.

Mass Spectrometric conditions for molar activity determination of [^18^F]FDHT: Mass spectrometric data were acquired in positive ion mode with the following ESI-MS parameters: Capillary Temperature 300 °C, Capillary Voltage 100 °C, Source Voltage Offset 20V, Source Voltage Span 30V, Source Gas Temperature 200 °C, ESI Voltage 3500V. Nitrogen was used as a desolvation gas. Quantification was done using a scan/SIM switching mode. The parameters for the scan mode was set as follows: scan range 50–800 *m*/*z*, scan time 100 ms, scan speed 7500 *m*/*z*/sec. In SIM mode dwell time was set at 300 ms for all analytes. Precursor ion (FDHT; calculated 308, found 350 [M + H + ACN], Verapamil; calculated 454, found 455 [M + H]) along with retention time 6.0 min was used to confirm the identity of the analyte in the sample.

### 3.2. Radiosynthesis of [^18^F]FFNP

Fluorine-18 labeled target water (7.4 GBq, 200 mCi) was diluted with 2 mL water and passed through an anion-exchange cartridge (Chromafix 30-PS-HCO_3_). The cartridge was washed with anhydrous acetonitrile (6 mL) and dried for 1 min under vacuum. The trapped [^18^F]fluoride from the Sep-Pak was manually eluted (0.5 mL/min) with triflate precursor (**1**, 3 mg) in 0.5 mL acetonitrile. The cartridge was flushed with 1 mL acetonitrile and collected in the same vial. The crude reaction mixture was diluted with 2 mL of water and purified by HPLC using a semi-preparative column (method B). The collected fraction (~15 min) was diluted with 15 mL water and [^18^F]FFNP was trapped by passing the solution through an activated Sep-Pak plus C-18 cartridge. The product was eluted with ethanol (0.6 mL) and saline (6 mL). The identity and purity of the product were confirmed by analytical HPLC (method A).

### 3.3. Radiosynthesis of [^18^F]FDHT

Fluorine-18 labeled target water (7.4 GBq, 200 mCi) was diluted with 2 mL water and passed through an anion-exchange cartridge (Chromafix 30-PS-HCO_3_). The cartridge was washed with anhydrous acetonitrile (6 mL) and dried for 1 min under vacuum. The trapped [^18^F]fluoride from the Sep-Pak was manually eluted (0.5 mL/min) with triflate precursor (**2**, 5 mg) in 0.5 mL tetrahydrofuran (THF). The cartridge was flushed with 1 mL acetonitrile, collected in the same vial and diluted with 10 mL of water. The mixture was passed through an activated Sep-Pak plus C-18 cartridge and the cartridge was washed with 6 mL of water. An aqueous solution of sodium borohydride (NaBH_4_, 40 mg in 1 mL) was slowly passed through the cartridge in 1 min, and the Sep-Pak was washed with 6 mL water followed by slowly passing 6N HCl (2 mL) in 2 min. The cartridge was further washed with 6 mL of water. The crude product was eluted with 2 mL of acetonitrile and diluted with 2 mL of water. The product was purified by HPLC using a semi-preparative column (method C). The collected fraction (~30 min) was diluted with 30 mL water and [^18^F]FDHT was trapped by passing the solution through an activated HLB light Sep-Pak. The Sep-Pak was first eluted with 0.2 mL ethanol, the eluted fraction was discarded. Next, the Sep-Pak was eluted with ethanol (0.2 mL) and water (0.8 mL) to the product vial. The identity and purity of the product were confirmed by analytical HPLC (method D) and LC/MS (method E).

### 3.4. Preparation of Calibration Curves for Molar Activity Determination

The molar activity was determined by LC-MS analysis. Briefly, a calibration curve in the range of 1–20 µg/mL (ppm) was prepared for FDHT using verapamil as an internal standard. The curves were fitted least squares linear regression method by measuring the peak area ratio of the analyte to the internal standard. The acceptable criterion for the calibration curve was a correlation coefficient (r2) of 0.99 or better, and that each back-calculated standard concentration must be within 20% deviation from the nominal value. Signal-to-noise (S/N) of the lowest calibration level was greater than 10.

### 3.5. In Vitro Studies

Saturation binding studies were performed to determine the K_d_ of [^18^F]FDHT and B_max_ with DU-145 AR+ cells [human prostate cancer cells transfected with androgen receptors (AR)] or cytosol preparations of DU-145 AR+ tumors [obtained from xenograft mouse models]. The tumor cytosols were prepared by homogenizing the tumor tissue in 5 to 10 volumes of homogenization buffer [10 mM Tris-HCl (pH 7.5) containing 1.5 mM EDTA] which was followed by ultracentrifugation (200,000× *g*; 1 h; 4 °C) of the homogenate. After centrifugation, cytosolic supernatants were collected and protein concentrations determined using the Bradford method [28].

To the tumor cytosols in tubes or plated DU-145 AR+ cells (3–4 × 10^5^cells/well; 1 d prior to the assay) increasing concentrations of [^18^F]FDHT (0.1–40 nM) were added to duplicate tubes; non-specific binding was determined by adding unlabeled FDHT (10^−6^ M) to another set of duplicates. After incubation (2 h, 4 °C), the bound [^18^F]FDHT was separated from the free as follows: (1) plated cells were washed with phosphate buffered saline (PBS), treated with trypsin, and collected in vials; or (2) to the cytosol in tubes DCC (0.4% dextran-coated charcoal) was added and pelleted by centrifugation from which the supernatants were collected. The bound radioactivity for these samples was determined by gamma counting (Perkin Elmer 2480 Wizard3, Shelton, CT). From the saturation studies, the K_d_ and B_max_ were determined from 6 to 8 concentrations of [^18^F]FDHT and analyzed using non-linear regression curve fitting [one-site specific binding, PRISM (version 5.04 Windows), GraphPad Software, San Diego, CA, USA].

## 4. Conclusions

Two well-known PET tracers [^18^F]FFNP and [^18^F]FDHT have successfully been prepared by “fluorination on Sep-Pak” method with moderate to high RCY in a short synthesis time (40–70 min). Both reduction of intermediate **3** and deprotection of reduced intermediate to prepare final [^18^F]FDHT were performed on a single Sep-Pak cartridge. Due to the poor UV absorption of FDHT the conventional molar activity, determination method was not applicable. We developed a method to determine the mass associated with [^18^F]FDHT by using LC-MS. The molar activities of [^18^F]FDHT determined from this newly developed method were found to be accurate and yielded K_d_ values (~2.5 nM) which were comparable to published values. This “fluorination on Sep-Pak” method might be applicable to other room temperature fluorine-18 labeling reactions.

## Figures and Tables

**Table 1:** Elution conditions from the Sep-Pak to prepare [^18^F]FFNP.

Tracer	Amount of Precursor 1 or 2 (mg)	Solvent (0.5 mL)	Eluted from the Sep-Pak (%) *^a^*
[^18^F]FFNP	1.5	Acetonitrile	42
	3	Acetonitrile	71–79 *^b^*
	5	Acetonitrile	76
		DMF	40
		DMSO	49
		THF	36

*^a^* Radiolabeling was performed with 0.37–0.74 GBq (10–20 mCi) of fluorine-18; *^b^ n* ≥ 6.

**Table 2:** Elution conditions from the Sep-Pak to prepare compound **3**, the intermediate for [^18^F]FDHT.

Compound	Amount of Precursor 1 or 2 (mg)	Solvent (0.5 mL)	Eluted from the Sep-Pak (%) *^a^*
3	1.5	Acetonitrile	14
	3	Acetonitrile	34
	5	Acetonitrile	52
		DMF	13
		DMSO	27
		THF	57 *^b^*

*^a^* Radiolabeling was performed with 0.37–0.74 GBq (10–20 mCi) of fluorine-18; *^b^ n* ≥ 6.
